# Development of the short form Iranian women childbirth experience questionnaire: a confirmatory factor analysis approach item reduction

**DOI:** 10.1186/s12884-023-05378-y

**Published:** 2023-01-20

**Authors:** Zohreh Shahhosseini, Roya Nikbakht, Zahra Motaghi, Monirolsadate Hosseini Tabaghdehi

**Affiliations:** 1 Sexual and Reproductive Health Research Center, Mazandaran University of Medical Sciences, Sari, Iran; 2 Department of Biostatistics and Epidemiology, Faculty of Health, Mazandaran University of Medical Science, Sari, Iran; 3 Department of Midwifery, School of Nursing and Midwifery, Shahroud University of Medical Sciences, Shahroud, Iran; 4 Department of Midwifery, Health Reproductive Research Center, Sari Branch, Islamic Azad University, Sari, Iran

**Keywords:** Childbirth, Experience, Confirmatory factor analysis, Item reduction

## Abstract

**Introduction:**

Considering that childbirth experience has short- and long-term effects on women’s lives, it is necessary to examine their delivery experiences. This study aimed to prepare the short form of a 52-item Iranian women’s childbirth experience questionnaire with seven factors: professional support, preparation, control, positive perception, baby, family support, and fear.

**Methods:**

This methodological research was conducted on women aged 15 to 49 years (*n* = 770) with uncomplicated vaginal delivery. The short form of the Iranian women’s childbirth experience questionnaire was prepared in four stages. The first stage was exploratory factor analysis, conducted on 250 samples, the second stage was confirmatory factor analysis which was performed on 260 samples, independent of the first stage, to report goodness and fit indices, and the third stage employed items from modification indices, expected parameter change, and standardized residual covariance, leading to the short form of Iranian women childbirth experience questionnaire. Finally, confirmatory factor analysis was run on 260 samples, independent of the previous two stages, to confirm the short form and compare it with the original questionnaire for psychometric analysis.

**Results:**

In the exploratory factor analysis stage, nine items with a factor load of less than 0.4 were removed, and the number of domains was reduced to five. The second stage showed that the questionnaire had a goodness of fit index. However, the third stage resulted in removing 11 overlapping items and making a short questionnaire with 33 items. Finally, the confirmatory factor analysis in the last stage showed appropriate goodness of fit for the short form of the Iranian women’s childbirth experiences questionnaire (𝛘^2^/df = 2.352, CFI = 0.881, PCFI = 0.750, RMSEA = 0.072, SRMR = 0.0862).

**Conclusion:**

The short form of the Iranian women’s childbirth experiences questionnaire enjoyed from an appropriate psychometric evaluation. It is recommended when applying the original questionnaire is not feasible due to lack of time.

**Supplementary Information:**

The online version contains supplementary material available at 10.1186/s12884-023-05378-y.

## Introduction

Childbirth experience is the women's personal feeling and interpretation of the birth process; it is a unique experience that evokes emotions, responses, and challenges in mothers. This experience is a lovely and exciting event for some women and a stressful and unpredictable experience for others [1].

This psychological and physiological process is under the effect of numerous factors, including the history of traumatic experiences, social support, expectations, history of mental illness, feelings during childbirth, pain perception, labor care, control [2], use of anesthetics, low Apgar score, and infant transfer to neonatal intensive care unit [3]. Besides, the childbirth experience is significantly affected by women's cultural perceptions, beliefs, fears, and cultural practices [4].

Women's delivery experiences affect the women's and infants' health, mother-infant relationship, family, and community. Therefore, the negative experience of childbirth results in increased anxiety [5], postpartum depression, increased suicidal ideation, decreased self-esteem, and decreased empowerment and self-efficacy of women. Correspondingly, it reduces the mother and child attachment, resulting in the mother's failure to breastfeed and less love for the child. However, it causes responsibility for parenthood, acceptance of the mother's role, and the appropriate relationship between couples at the family level. It affects the rate of childbearing, fertility, and cesarean section and reduces abortion at the community level [6].

Given the importance of childbirth experiences, it is necessary to plan to promote pleasurable childbirth experiences. The first step is to examine the current situation, which requires instruments appropriate to the culture of the community. There are various instruments to measure childbirth experiences. Most of them evaluate merely one dimension of childbirth experiences such as women’s perception of their childbirth experience [7], support and control in birth [8], patient perceptions and control of the childbirth environment [9], and fear during the process of labor [10].

The Turkish multidimensional 23-item childbirth experience questionnaire 2.0. [11], has been validated in a sample of Iranian primiparous women [12]. As the perception of the childbirth experience is highly personalized, and the childbirth experiences of primiparous women are different from multiparous ones [13–15], thus introducing an instrument to assess the childbirth experiences of multiparous women seems necessary.

Hosseini et al. (2020) have developed a questionnaire related to vaginal delivery experiences. It includes 52 items with seven subscales: professional support, preparation, control, positive perception, baby, family support, and fear. According to this instrument, those who score higher have more positive experiences [16]. Since this instrument is completed within 12 h to 42 days after the delivery and the minimum required time to complete is 30 min, this may be beyond their tolerance. Therefore, to increase their attention while completing the questionnaire, the research team sought to provide an instrument with fewer items, identical to the original one in terms of conceptual content and psychometrics.

## Method

This study aimed to prepare the short form of the Iranian women's childbirth experiences questionnaire for those with vaginal delivery to show the original questionnaire's conceptual model as much as possible.

### Data

This methodological study was conducted in four stages from June to December 2021 in Mazandaran, Iran. Sampling was done based on the convenience sampling method among women aged 15 to 49 with an uncomplicated vaginal delivery within 12 h to two months after delivery in health centers and hospitals.

Comrey has recommended the sufficient sample size in factor analysis be 100 = poor, 200 = fair, 300 = good, 500 = very good, 1000 and higher = excellent [17] In this study, 770 people completed the questionnaire.

### Ethical considerations

All the procedures performed in this study that involved human subjects were in full compliance with the ethical standards of the institutional and national research committee and the 1964 Helsinki Declaration and its later amendments or comparable ethical standards. The Ethics Committee of the Mazandaran University of Medical Sciences, Sari, Iran, has approved this study (Code:IR.MAZUMS.REC.1400.467). Written informed consent was obtained from all participants.

### Measures

#### Demographic characteristics form

This form consist of information about the participant's age, education, occupation, and the number of pregnancies.

#### Iranian Women's Childbirth Experience Questionnaire (IWCEQ)

This questionnaire was designed by Hosseini Tabaqdehi et al. in a sequential exploratory study to evaluate the childbirth experiences of Iranian women after a vaginal delivery. In the first stage of this questionnaire, a qualitative study with a content analysis approach was used to explain the concept and dimensions of women’s childbirth experience. Then, in the second stage, a quantitative study with an inductive-deductive approach was designed and its psychometric properties were evaluated. Finally, a 52-item tool was designed. It included seven subscales based on the five-point Likert scale, including strongly disagree = 1, disagree = 2, undecided = 3, agree = 4, and strongly agree = 5. The subscales include professional support (items 1–15), preparation (items 16–22), positive perception (items 23–32), baby (items 33–37), family support (items 38–42), control (items 43–49), and fear (items 50–52). The items 49, 50, 51, and 52 were coded inverse [16].

### Statistical analysis

According to Goetz et al., the rigorous methodology was employed in four stages to prepare the short form of a questionnaire [18].

#### Stage 1

The researchers established Exploratory Factor Analysis (EFA) using the principal axis factoring method with varimax rotation. First, the questionnaire was given to 250 women, and the construct validity of the questionnaire was assessed. To guarantee sufficient sample size, KMO was used; its value should be greater than 0.6 for the questionnaire. Moreover, Bartlett's test result needs to be significant [19].

#### Stage 2

In the second stage, the researchers performed Confirmatory Factor Analysis (CFA) on 260 samples using Amos software version 24. They obtained five factors from the 52-item Iranian women's childbirth experiences questionnaire using EFA. Then, the questionnaire was examined using a CFA.

The principal basis of data analysis was based on CFA with a significance level of 0.05. The reported indices were Goodness Of Fit (GOF), each of which should be in a particular range to accept that the model has a good fit. The changes in the df/χ^2^ index range between 1 and 5. Another index used for a good fit is the Parsimony Comparative Fit Index (PCFI); if its value is > 0.5, it indicates the proper fit of the model. Moreover, the Comparative Fit Inde2 (CFI) should be greater than 0.9. Another index is the Root Mean Square Error of Approximation (RMSEA) index; if it is between 0.05 and 0.08, the model has a good fit. The last index is the Standardized Root Mean Square Residual (SRMR); if it is smaller than 0.1, the model has a good fit [20].

#### Stage 3

At this stage, the researchers selected the items to preserve the psychometric properties and content validity. They also used the correlation and overlap index to remove the items. The selection criteria for these indices were Modification Indices (MI) (> 10), Expected Parameter Change (EPC) values > 0.2, and large standardized residual covariance (> 0.2) [21].

#### Stage 4

Finally, a CFA was conducted for the short version of the questionnaire with 260 samples, independent of the samples in the previous stages, to determine whether the short form enjoys the same GOF as the original questionnaire.

## Results

Most participants aged 26 to 30 years (*n* = 278, 36.1%), 411 (53.4%) were primiparas, 245 (31.8%) were high school drop-outs, 315 (40.9%) had diplomas, and 210 (27.3%) held university degrees.

### Stage 1 results

The KMO index was applied to determine sample size adequacy. The overall KMO value was 0.90, and Bartlett's test was significant (*P*-value < 0.001). The KMO value was checked for each item; items with a KMO < 0.5 should be removed. However, the KMO values ranged from 0.54 to 0.96, so all the items remained.

The EFA was conducted using the principal axis factoring and varimax rotation methods considering five domains identified by the scree plot. At this stage, nine items were deleted with a factor load < 0.4). The remaining items in the five domains explained 46.45% of the variance. The results are attached in Supplementary Table 1.

- 1. Staff support: items 1 to 15 and 48 (*n* = 16)
- 2. Competence: items 16–18, 20, 24, 26, 44, 47 (*n* = 8)
- 3. Baby (bonding): items 19, 33–37 (*n* = 6)
- 4. Empowerment: items 23, 25, 27, 28–32 (*n* = 8)

- 5. Family support: items 38–42 (*n *= 5)

Items 21, 22, 43, 45, 46, 49, 50, 51, and 52 had a factor load < 0.4, so they were omitted.

### Stage 2 results

At this stage, the principal basis of data analysis was based on CFA, with a significant level of 0.05 in all tests.

Table 1 demonstrates the correlation matrix (r) of the research variables. According to the results reported in Table 1, the correlation between all factors is significant.

**Table 1:** Correlation between research variables

	**1**	**2**	**3**	**4**	**5**
Factor 1: Staff support	1				
Factor 2: Competence	0.44^b^	1			
Factor 3: Baby bonding	0.37^b^	0.32^b^	1		
Factor 4: Empowerment	0.50^b^	0.45^b^	0.49^b^	1	
Factor 5: Family support	0.36^b^	0.27^b^	0.50^b^	0.40^b^	1

^a^Correlation is significant at the .05 level (two-tailed)

^b^Correlation is significant at the .01 level (two-tailed)

**Fig. 1:**
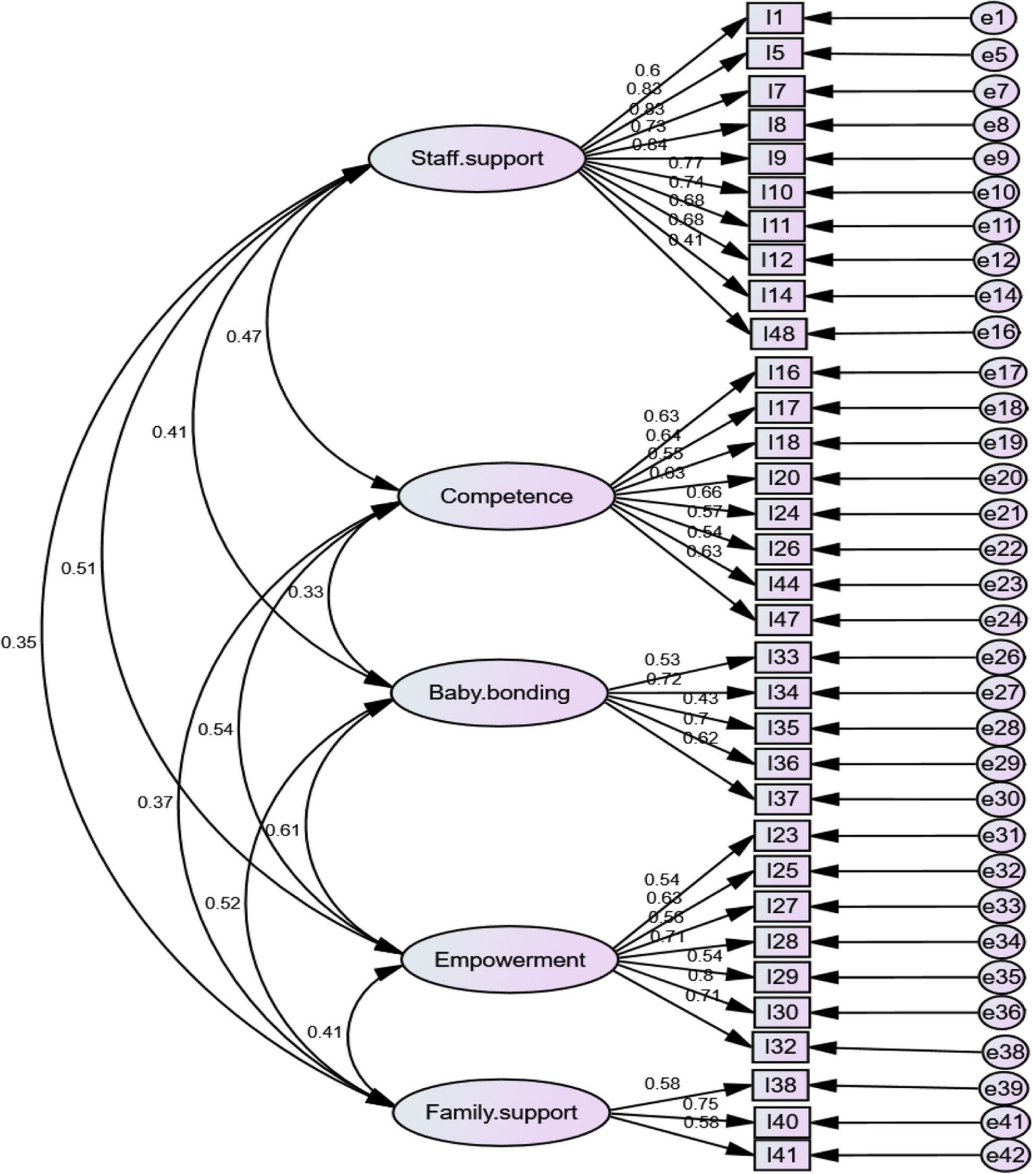
Final research model and its parameters using standardized data

The initial and final models were investigated by structural equations using Amos 24.0 software and the maximum likelihood (ML) model. Supplementary Table 2 reports the GOF indices for both models. Moreover, Fig. 1 illustrates the final model.

In the first measurement model, since item 19 of the attachment had a coefficient < 0.4, it was eliminated, and CFA was performed step by step without it (Supplementary Table 2).

### Stage 3 results

At this stage, the researchers identified the items with high correlation coefficients and removed one of them that had a more negligible effect on improving the GOF indices. Moreover, one of the items with MI > 10 and EPC > 0.2 was excluded (Table 2). Finally, the GOF indices of the final model were reported. According to Supplementary Table 2, all indices are in the acceptable range. Therefore, the model has enjoyed from a GOF for the data, and the relevant factors are approved.

**Table 2:** Iranian women's childbirth experience questionnaire: item content and reasons for item omission

Items	Reason for item omission
Staff support	
1. The midwife was friendly with me	Retained
2. I had a good interaction with my midwife	Excluded due to overlap with item 1 (MI = 58.03, EPC = 0.40, *r* = 0.70)
3. The midwife provided the training to control delivery during childbirth and labor	Excluded due to overlap with item 8 (MI = 14.03, EPC = 0.19, *r* = 0.64)
4. The delivery room staff had a good relationship with me during the delivery process	Excluded due to conceptual overlap with item 5, strong empirical overlap (*r* = 0.70)
5. The midwife understood my needs and wishes	Retained
6. The midwife treated me politely and respectfully	Excluded due to conceptual overlap with item 5, strong empirical overlap (*r* = 0.73)
7. The midwife spent enough time with me	Retained
8. The midwife informed me of what would happen during the delivery	Retained
9. The midwife took good care of me	Retained
10. The midwife in the delivery room was calm	Retained
11. The midwife encouraged me to adapt to childbirth and continue the process	Retained
12. The midwife considered my wishes for delivery	Retained
13. The delivery environment was safe and comfortable	Excluded due to overlap with item 14 (MI = 23.32, EPC = 0.27, *r* = 0.60)
14. The staff warmly welcomed me as I entered the delivery ward	Retained
15. I received the appropriate, timely, and necessary services in the delivery department	Excluded due to overlap with item 14 (MI = 10.17, EPC = 0.19, *r* = 0.50)
48. I tolerated labor pain when the midwife talked to me while working	Retained
Competence	
16. I thought I had the ability and power of natural childbirth	Retained
17. I would like to experience natural childbirth	Retained
18. In the end, the pain of natural childbirth is sweet	Retained
20. I felt happy during labor	Retained
24. I have good memories of childbirth	Retained
26. The delivery was not as painful as I thought	Retained
44. I could comment on the natural childbirth process and related decisions	Retained
47. I tolerated the pain of labor more efficiently by relying on good thoughts	Retained
Baby bonding	
19. I planned to get pregnant at the right time	Excluded at Step 2 due to low standardized regression coefficient, 0.28 (less than 0.4)
33. I was very eager to see the baby during labor	Retained
34. I was encouraged to embrace the baby immediately after delivery	Retained
35. Immediately after giving birth, I heard my baby crying	Retained
36. Immediately after giving birth, I was able to see my baby for the first time in a satisfactory way	Retained
37. I kept my baby for the first time as I wanted	Retained
Empowerment	
23. I had healthy and safe childbirth	Retained
25. I felt more responsible after giving birth	Retained
27. I felt light and comfortable after giving birth	Retained
28. After giving birth, I realized my inner strength	Retained
29. I felt empowered after childbirth	Retained
30. I felt successful with the delivery	Retained
31. I felt independent and self-sufficient during childbirth	Excluded due to conceptual overlap with item 28, strong empirical overlap (*r* = 0.66)
32. My self-confidence increased with childbirth	Retained
Family support	
38. The support and presence of my parents were helpful during the delivery	Retained
39. The presence of my husband made me feel strong	Excluded due to conceptual overlap with item 40, strong empirical overlap (*r* = 0.70)
40. My husband's support during the delivery was helpful	Retained
41. My husband encouraged me to have a natural delivery during my pregnancy	Retained
42. My family encouraged me to have a natural delivery during my pregnancy	Excluded due to overlap with Item 41 (MI = 29.79, EPC = 0.30, *r* = 0.52)
Deleted items before CFA (loading < 0.4)	
21. I was very hopeful during the delivery	Excluded due to dropped dimension
22. I was familiar with the delivery environment before childbirth	Excluded due to dropped dimension
43. I believed that after natural childbirth, I would be able to do my baby's work independently	Excluded due to dropped dimension
45. I have gained the necessary knowledge about childbirth using various sources	Excluded due to dropped dimension
46. ​​Knowing the labor pains and techniques of dealing with childbirth, I was ready for labor	Excluded due to dropped dimension
49. The hectic environment reduced my tolerance	Excluded due to dropped dimension
50. I was afraid of my child's hurting and death	Excluded due to dropped dimension
51. I was afraid of labor pains	Excluded due to dropped dimension
52. I was worried and anxious during labor	Excluded due to dropped dimension

### Stage 4 results

**Fig. 2:**
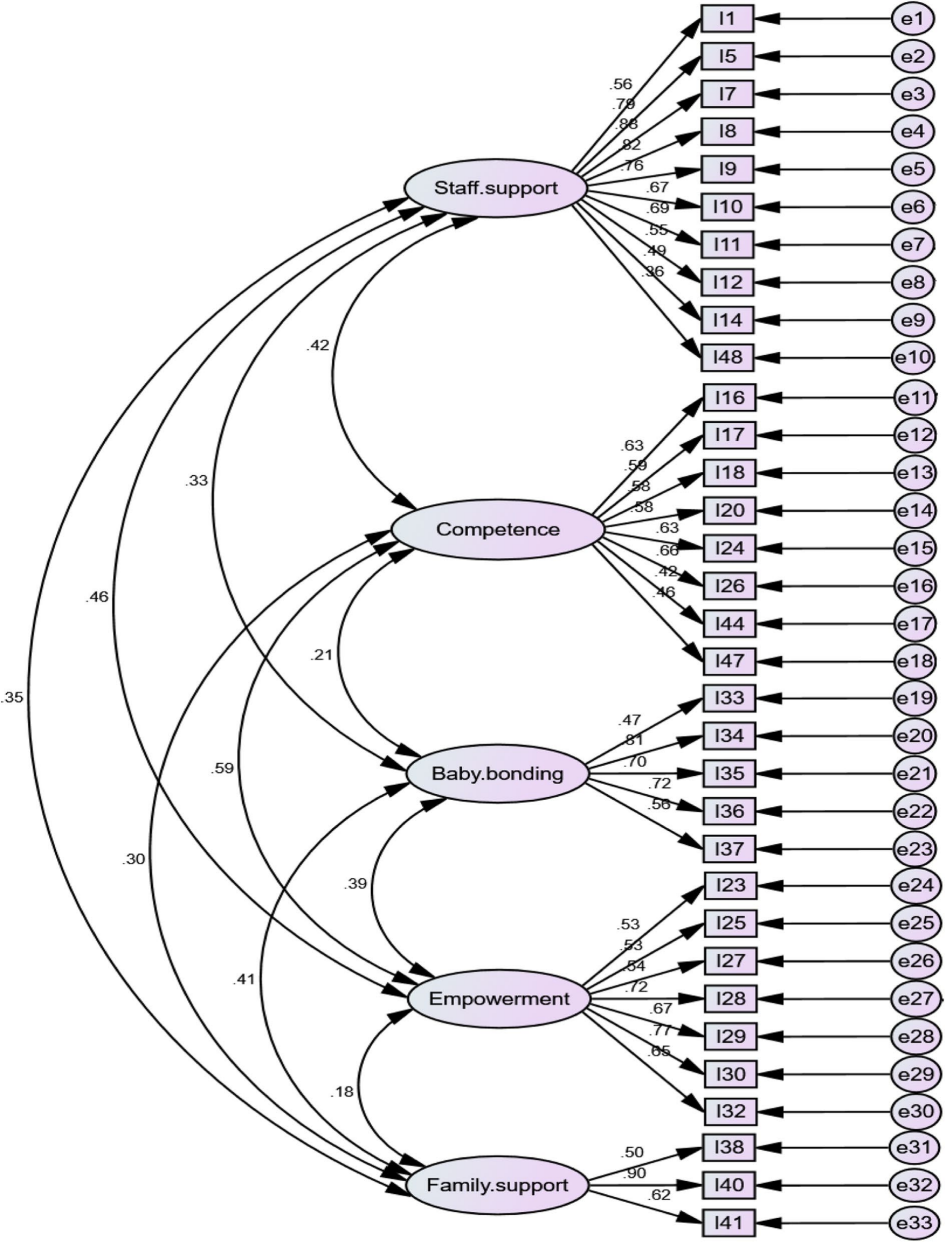
Final research model and its parameters using standardized data (four stage factor analysis)

The principal basis of data analysis in this stage was based on CFA, with a significant level of 0.05 in all tests. All indices were in the acceptable range (χ^2^/df = 2.352, CFI = 0.881, PCFI = 0.750, RMSEA = 0.072, SRMR = 0.0862). Therefore, the model has a good fit, and the short form of the questionnaire is approved (Fig. 2).

 Table 3 illustrates the standardized effects of factors that are correlation coefficients. The reported *P*-values reveal that all relationships are significant (*P*-value < 0.05).

**Table 3:** Results for standardized effects of variables

Variables	Correlation Estimate	Covariance Estimate	SE	*P*-value
Staff support ↔ Competence	0.42	0.087	0.019	< 0.0001
Staff support ↔ Baby bonding	0.33	0.036	0.010	< 0.0001
Staff support ↔ Empowerment	0.46	0.077	0.017	< 0.0001
Staff support ↔ Family support	0.35	0.056	0.015	< 0.0001
Competence ↔ Baby bonding	0.21	0.032	0.012	0.011
Competence ↔ Empowerment	0.59	0.134	0.026	< 0.0001
Competence ↔ Family support	0.30	0.065	0.020	< 0.0001
Baby bonding ↔ Empowerment	0.39	0.047	0.012	< 0.0001
Baby bonding ↔ Family support	0.41	0.047	0.012	< 0.0001
Empowerment ↔ Family support	0.18	0.032	0.014	0.026

## Discussion

This study has presented the stages of preparing the short form of Iranian women's childbirth experience questionnaire based on Goetz psychometric and conceptual criteria [18]. This process resulted in a questionnaire with five subscales: staff support, competence, baby bonding, empowerment, and family support.

The researchers examined the psychometric properties of the questionnaire in terms of model fit (factor analysis), item fit indices, and reliability (all values > 0.70). The short form demonstrated a good fit with the data without applying the correlated errors; these results illustrate the favorable psychometric status of the shortened Iranian women's childbirth experience questionnaire. For the short form of this questionnaire, we decided not to reduce it to fewer items because the main aspects (different aspects and high specificity) of this questionnaire would be spoiled. It was almost impossible to select fewer items without compromising the level of information.

This study reduced the number of subscales to five, and the "fear" domain was omitted. Some studies have shown that ignorance, unrealistic expectations, and lack of feeling of security cause fear [22]. This fear increases the negative experiences of childbirth examined in other domains of these issues in the short form of the questionnaire. The five-subscale short form of the questionnaire includes items for all six remaining subscales. However, some items were removed because they overlapped, and the research team renamed the remaining domains according to the related questions.

### Staff support subscale

In this study, items 1 and 2 conceptually overlapped items 4 and 5. The results demonstrated that the service providers created appropriate interactions using proper verbal and non-verbal communication. Moreover, they could understand the needs of women. In this regard, Olubunmi et al. have shown that women have unique needs during labor and delivery, and the service providers must understand them through an appropriate relationship. They should take action to meet these needs considering the acceptable cultural and social norms [22, 23].

Furthermore, items 3 and 8 conceptually overlapped. The results revealed that providing information and increasing the women's awareness helped them better control childbirth during the delivery process because it created realistic expectations, which led to a better understanding and pleasant delivery experience [23, 24].

Correspondingly, items 13, 14, and 15 conceptually overlapped, and all were related to the psychosocial factors affecting the childbirth experience. In line with this issue, Goldkuhl et al. have demonstrated that interaction, organizational factors, and physical environment are related to the delivery environment [25]. As a result, creating the right environment for childbirth reduces women's stress during childbirth, and they will have physiological childbirth and a better understanding of delivery. Therefore, the service provider can play an efficient role in creating a feeling of security, control, and knowledge of delivery through proper interaction, optimal implementation of organizational policy, and providing a safe delivery environment [23].

### Competence subscale

In this subscale, items 16, 17, 18, and 20, items 24 and 26, and items 44 and 47 were related to preparation, positive perception, and control subscales, respectively. In this study, the EFA and CFA integrated them and created the concept of competence. Competence is the application of knowledge, skills, attitudes, and values in a given situation [26]. In childbirth, it is the women's understanding of various delivery factors, such as knowledge, motivation, skills, and other factors.

### Family support subscale

Item 39 was deleted due to the high conceptual correlation with item 40, indicating the efficient role of the spouse in the labor and delivery stages. Moreover, item 42 overlapped item 41 in terms of concept; i.e., both showed encouraging the woman for vaginal delivery by her companions.

### Empowerment subscale

The items existed in the original form of the article. Item 31 conceptually overlapped item 28, and both showed concepts of empowerment. Empowerment includes a sense of satisfaction, contentment, independence, improved interaction with others and the environment, and better adaptation to physical and psychological changes [27]. According to the World Health Organization, empowerment is a process by which individuals will have more control over factors that affect health, such as decisions, lifestyles, and activities. According to the above definitions, high capability indicates pleasant delivery experiences, which can assess one's delivery experiences by evaluating items in this dimension.

### Bonding subscale

All items in this subscale remained in the short form of the questionnaire. These items demonstrate the importance of early contact between mother and baby after birth or bonding. In line with this issue, Seefeld et al. have revealed that bonding reflects the childbirth experience. If one has a positive delivery experience, good bonding happens. Hence, the childbirth experience can be determined by knowing the bonding [28]. Since mother–child bonding is a risk factor for impaired child's emotional, behavioral, and cognitive development [29], we can create an appropriate emotional mother-baby bonding and enhance the child's emotional, behavioral, and cognitive growth through a positive delivery experience.

### Strengths and weaknesses

One of the limitations of this study is judging the conceptual content based on the authors' opinions. Other limitations include using statistical methods to shorten the instrument and not using the viewpoint of a gynecologist. On the other hand, this study has used a large sample size and analyzed the data in different stages on samples independent of each stage.

## Conclusion

The short form of the Iranian women's childbirth experience questionnaire examines women's delivery experiences from several subscales. Compared to the original form of the instrument, the conceptual and psychometric features are preserved; therefore, the researchers recommend using it in cases when applying the original questionnaire is not feasible due to lack of time.

## Supplementary Information

**Additional file 1: Supplementary Table 1. **Exploratory Analysis Factor Results.

**Additional file 2: Supplementary Table 2. **The goodness of fit indices after amodel modification step.

## Data Availability

Datasets used and analyzed during this study are available from the corresponding author on reasonable request.
