# Supplementary material for: Development of the short form Iranian women childbirth experience questionnaire: a confirmatory factor analysis approach item reduction
Source: BMC Pregnancy Childbirth. 2023 Jan 20;23:48. doi: 10.1186/s12884-023-05378-y (PMC9854137; doi:10.1186/s12884-023-05378-y)
Supplement: Supplementary file 1 — Additional file 1: Supplementary Table 1. Exploratory Analysis Factor Results. [file 12884_2023_5378_MOESM1_ESM.docx]

**Supplementary Table 1. Exploratory Analysis Factor Results**

| **Row** | **Question** | **Loadings** | | | | |
| --- | --- | --- | --- | --- | --- | --- |
|  |  | **F1** | **F2** | **F3** | **F4** | **F5** |
| 1 | The midwife was friendly with me. | 0.68 |  |  |  |  |
| 2 | I had a good interaction with my midwife. | 0.58 |  |  |  |  |
| 3 | During childbirth and labor, the midwife provided the necessary training to control childbirth. | 0.65 |  |  |  |  |
| 4 | The delivery room staff had a good relationship with me during the delivery process. | 0.77 |  |  |  |  |
| 5 | The midwife understood my needs and wishes. | 0.83 |  |  |  |  |
| 6 | The midwife treated me politely and respectfully. | 0.82 |  |  |  |  |
| 7 | The midwife spent enough time with me. | 0.84 |  |  |  |  |
| 8 | The midwife informed me of what would happen during the delivery. | 0.77 |  |  |  |  |
| 9 | The midwife took good care of me. | 0.86 |  |  |  |  |
| 10 | The midwife in the delivery room was calm. | 0.81 |  |  |  |  |
| 11 | The midwife encouraged me to adapt to childbirth and continue the process. | 0.73 |  |  |  |  |
| 12 | The midwife considered my wishes for delivery. | 0.62 |  |  |  |  |
| 13 | The delivery environment was safe and comfortable. | 0.61 |  |  |  |  |
| 14 | The staff warmly welcomed me as I entered the delivery ward. | 0.64 |  |  |  |  |
| 15 | I received the appropriate, timely, and necessary services in the delivery department. | 0.59 |  |  |  |  |
| 16 | I thought I had the ability and power of natural childbirth. |  |  |  |  | 0.49 |
| 17 | I would like to experience natural childbirth. |  |  |  |  | 0.66 |
| 18 | In the end, the pain of natural childbirth is sweet. |  | 0.43 |  |  | 0.55 |
| 19 | I planned to get pregnant at the right time |  | 0.44 |  |  |  |
| 20 | I felt happy during labor. |  |  |  |  | 0.52 |
| 21 | I was very hopeful during the delivery |  | | | | |
| 22 | I was familiar with the delivery environment before childbirth |  | | | | |
| 23 | I had a healthy and safe childbirth |  |  | 0.41 |  |  |
| 24 | I have good memories of childbirth |  |  |  |  | 0.59 |
| 25 | I felt more responsible after giving birth |  | 0.69 | 0.40 |  |  |
| 26 | The delivery was not as painful as I thought |  |  |  |  | 0.51 |
| 27 | I felt light and comfortable after giving birth | 0.42 |  |  | 0.49 |  |
| 28 | After giving birth, I realized my inner strength |  | 0.49 | 0.61 |  |  |
| 29 | I felt empowered after childbirth |  |  | 0.62 |  |  |
| 30 | I felt successful with the delivery |  |  | 0.67 |  |  |
| 31 | I felt independent and self-sufficient during childbirth |  |  | 0.69 |  |  |
| 32 | My self-confidence increased with childbirth |  |  | 0.60 |  |  |
| 33 | I was very eager to see the baby during labor |  | 0.61 |  |  |  |
| 34 | I was encouraged to embrace the baby immediately after delivery |  | 0.58 |  |  |  |
| 35 | Immediately after giving birth, I heard my baby crying |  | 0.60 |  |  |  |
| 36 | Immediately after giving birth, I was able to see my baby for the first time in a satisfactory manner |  | 0.54 |  |  |  |
| 37 | I kept my baby for the first time as I wanted. |  | 0.44 |  |  |  |
| 38 | The support and presence of my parents were helpful during the delivery |  |  |  | 0.41 |  |
| 39 | The presence of my husband made me feel strong. |  | 0.46 |  | 0.42 |  |
| 40 | My husband's support during the delivery was helpful. |  |  |  | 0.56 |  |
| 41 | My husband encouraged me to have a natural delivery during my pregnancy. |  |  |  | 0.53 |  |
| 42 | My family encouraged me to have a natural delivery during my pregnancy. |  |  |  | 0.52 |  |
| 43 | I believed that after natural childbirth, I would be able to do my baby's work on my own |  | | | | |
| 44 | I could comment on the natural childbirth process and related decisions |  |  |  |  | 0.40 |
| 45 | I have gained the necessary knowledge about childbirth using various sources |  | | | | |
| 46 | ​​Knowing the labor pains and techniques of dealing with childbirth, I was ready for labor. |  | | | | |
| 47 | I tolerated the pain of labor more efficiently by relying on good thoughts |  |  |  | 0.471 | 0.466 |
| 48 | I tolerated labor pain when the midwife talked to me while working | 0.60 |  |  |  |  |
| 49 | The hectic environment reduced my tolerance. |  | | | | |
| 50 | I was afraid of my child's hurting and death. |  | | | | |
| 51 | I was afraid of labor pains |  | | | | |
| 52 | I was worried and anxious during labor. |  | | | | |
