# Supplementary material for: Development of the short form Iranian women childbirth experience questionnaire: a confirmatory factor analysis approach item reduction
Source: BMC Pregnancy Childbirth. 2023 Jan 20;23:48. doi: 10.1186/s12884-023-05378-y (PMC9854137; doi:10.1186/s12884-023-05378-y)
Supplement: Supplementary file 2 — Additional file 2: Supplementary Table 2. The goodness of fit indices after amodel modification step. [file 12884_2023_5378_MOESM2_ESM.docx]

**Supplementary Table 2. The goodness of fit indices after a model modification step**

| **Stage** |  | **𝛘^2^/df** | **CFI** | **PCFI** | **RMSEA** | **SRMR** |
| --- | --- | --- | --- | --- | --- | --- |
| 1 | Initial model of measurement | 2.386 | 0.785 | 0.739 | 0.073 | 0.0808 |
| 2 | Omission of item 19 | 2.434 | 0.787 | 0.740 | 0.074 | 0.0801 |
| 3 | Omission of item 2 | 2.314 | 0.806 | 0.756 | 0.071 | 0.0771 |
| 4 | Omission of item 3 | 2.270 | 0.815 | 0.763 | 0.070 | 0.0772 |
| 5 | Omission of item 13 | 2.238 | 0.823 | 0.769 | 0.069 | 0.0770 |
| 6 | Omission of item 15 | 2.227 | 0.830 | 0.773 | 0.069 | 0.0767 |
| 7 | Omission of item 42 | 2.167 | 0.843 | 0.783 | 0.067 | 0.0750 |
| 8 | Omission of item 6 | 2.142 | 0.846 | 0.784 | 0.066 | 0.0749 |
| 9 | Omission of item 4 | 2.115 | 0.849 | 0.785 | 0.066 | 0.0751 |
| 10 | Omission of item 31 | 2.084 | 0.854 | 0.787 | 0.065 | 0.0747 |
| 11 | Omission of item 39 | 2.083 | 0.854 | 0.784 | 0.065 | 0.0724 |
| 12 | e24↔e16 | 2.035 | 0.861 | 0.789 | 0.063 | 0.0717 |
| 13 | e1↔e31 | 2.001 | 0.865 | 0.791 | 0.062 | 0.0715 |
| 14 | e14↔e35 | 1.981 | 0.868 | 0.793 | 0.062 | 0.0712 |
| 15 | e29↔e36 | 1.955 | 0.872 | 0.795 | 0.061 | 0.0709 |
| 16 | e18↔e26 | 1.931 | 0.876 | 0.796 | 0.060 | 0.0707 |
| 17 | e20↔e22 | 1.904 | 0.880 | 0.798 | 0.059 | 0.0698 |
| 18 | e18↔e41 | 1.880 | 0.883 | 0.799 | 0.058 | 0.0697 |
| 19 | e5↔e19 | 1.859 | 0.886 | 0.800 | 0.058 | 0.0694 |
| 20 | e11↔e26 | 1.839 | 0.889 | 0.801 | 0.057 | 0.0690 |
